# Supplementary material for: Neutrophil extracellular traps arm DC vaccination against NPM-mutant myeloproliferation
Source: eLife. 2022 Apr 26;11:e69257. doi: 10.7554/eLife.69257 (PMC9068207; doi:10.7554/eLife.69257)
Supplement: Supplementary file 1. [file elife-69257-supp1.docx]

| **Supplementary File 1**  Antibodies for flow cytometry | |  |  |
| --- | --- | --- | --- |
| **Antibody** | **Fluorochrome** | **Clone** | **Supplier** |
| **Flow cytometry** | | | |
| **CD11b** | PerCP-Cy5.5 | M1/70 | BD |
| **GR-1** | APC-Cy7 | RB6-8C5 | BD |
| **CD117 (c-Kit)** | PE-Cy7 | 2B8 | BD |
| **CD45** | APC--Cy7 | 30-F11 | BD |
| **CD45.1** | BV510 | A20 | BD |
| **CD45.2** | BV605 | 104 | BD |
| **CD4** | BV510 | RM4-5 | BD |
| **CD8** | PE-Cy7 or BV605 | 53-6.7 | BD |
| **CD44** | PerCP-Cy5.5 | IM7 | BD |
| **CD62L** | PE-Cy7 | MEL-14 | BD |
| **OX40** | BV711 | OX40 | BD |
| **CD41** | APC | eBioMWReg30 | e-Biosciences |
| **CD150** | BV605 | TC15-12F12.2 | Biolegend |
| **CD34** | FITC | RM34 | e-Biosciences |
| **Lineage markers:** |  |  |  |
| **CD3** | PE | 145-2C11 | e-Biosciences |
| **B220** | PE | RA3-6B2 | BD |
| **Ter119** | PE | TER-119 | BD |
| **CD11b** | PE | M1/70 | e-Biosciences |
| **CD11c** | PE | N418 | e-Biosciences |
| **Gr-1** | PE | RB&-8C5 | e-Biosciences |
